# Iridoschisis and keratoconus in a patient with severe allergic eye disease and compulsive eye rubbing: a case report

**DOI:** 10.1186/s13256-016-0914-7

**Published:** 2016-05-25

**Authors:** Imran H. Yusuf, John F. Salmon

**Affiliations:** The Oxford Eye Hospital, West Wing, John Radcliffe Hospital, Headley Way, , Headington Oxford, OX3 9DU UK

**Keywords:** Iridoschisis, Keratoconus, Allergic eye disease, Eye rubbing, Open drainage angle

## Abstract

**Background:**

Iridoschisis is a rare disorder characterized by splitting of the anterior and posterior iris stroma, resulting in disintegrated iris fibrils which float freely in the anterior chamber. We report an exceptional case of bilateral iridoschisis occurring in conjunction with keratoconus and severe allergic eye disease.

**Case presentation:**

A 24-year-old white man had had periocular contact dermatitis and allergic eye disease from the age of 3 years. He was allergic to grass, animal hair, and pollen and worked grooming horses. He compulsively rubbed his eyes. There was no history of previous blunt trauma to either eye. There were signs of bilateral iridoschisis and keratoconus with allergic conjunctivitis, all of which were more severe in his right eye. An open drainage angle was identified bilaterally on gonioscopy, excluding primary angle closure. There was no evidence of glaucoma in either eye.

**Conclusions:**

There are two previous cases reporting the combination of iridoschisis and keratoconus, but no clear common etiology has been identified. In this case there was no evidence of angle closure but there were signs of allergic conjunctivitis. This amalgamation of signs might be explained on the basis of habitual eye rubbing. Treating the allergic eye disease has attenuated this behavior.

## Background

Iridoschisis is a rare disorder characterized by localized cleavage of the anterior and posterior iris stroma [1]. The anterior leaf of the iris is atrophic and has disintegrated into fibrils, which float freely in the anterior chamber. A clear pathogenesis of iridoschisis has not been identified, although partial or complete angle closure in association with reduced axial length and shallow anterior chamber depth is commonly found [2, 3]. We report an exceptional case of bilateral iridoschisis, keratoconus, and severe allergic eye disease in a patient with an open drainage angle and a deep anterior chamber in both eyes. This case suggests the intriguing possibility that his ophthalmic pathology may be explained on the basis of habitual eye rubbing.

## Case presentation

A 24-year-old right-handed white man presented with a 21-year history of compulsive eye rubbing secondary to periocular atopic dermatitis and allergic conjunctivitis. He was allergic to animals, grass, and pollen and worked grooming horses. Topical emedastine 0.05 % drops, periocular 1 % hydrocortisone, and oral antihistamines were used to control his symptoms. His general health was excellent.

**Fig. 1:**
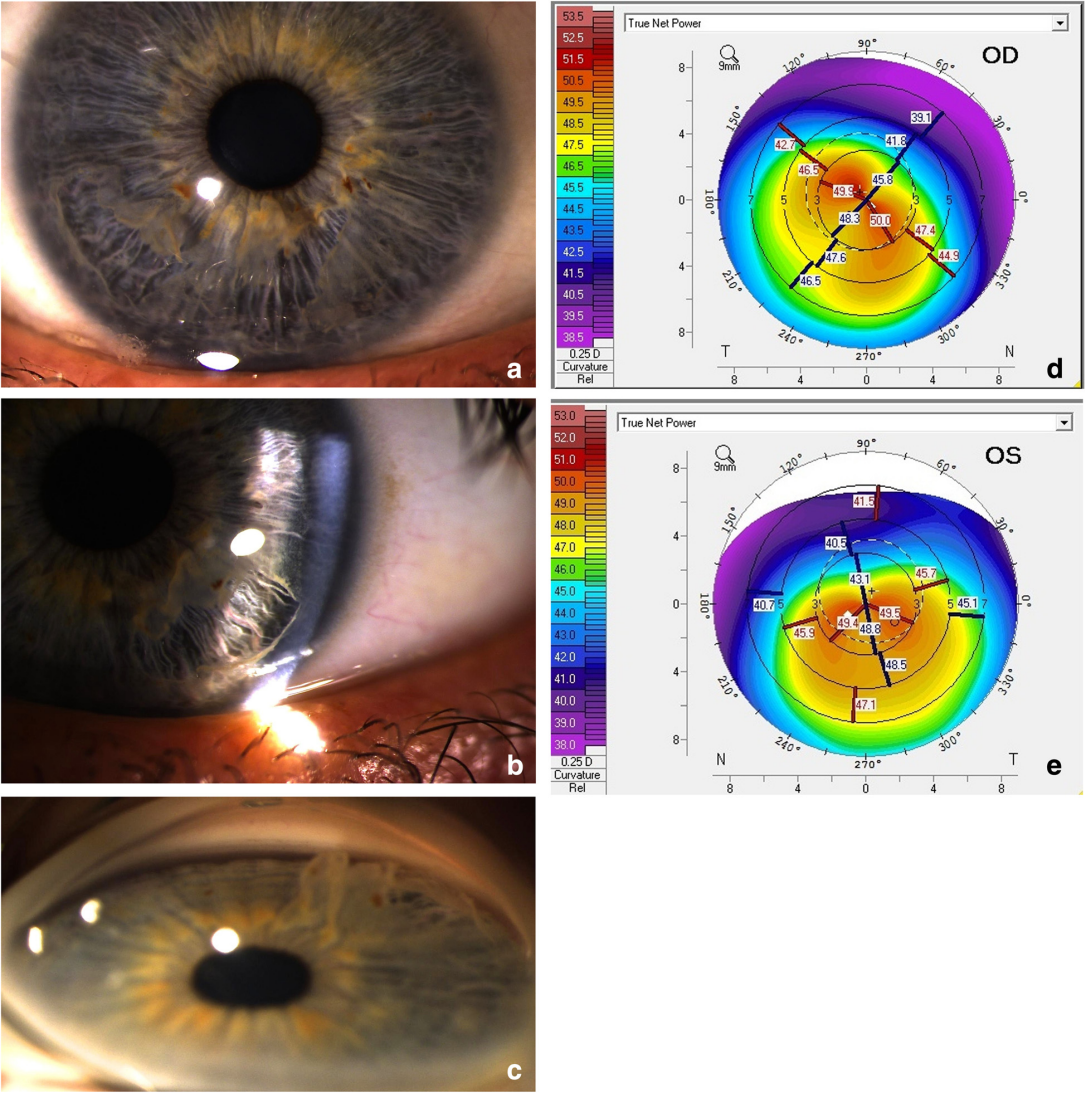
Anterior segment photograph of right (**a**) and left eyes (**b**) demonstrating asymmetrical inferior iridoschisis worse on the right, maximal in the inferotemporal quadrant. Anterior iris stroma can be identified protruding into the deep anterior chamber. Gonioscopy reveals an open drainage angle with a ciliary body band visible in the inferior drainage angle of the left eye (**c**). Note the protrusion of the anterior iris stroma into the anterior chamber on gonioscopy. Oculus Pentacam corneal topography (anterior surface) demonstrates bilateral keratoconus, worse on the right (**d**) than the left (**e**) with apical decentration. Central corneal thickness was 449 microns right eye and 429 microns left eye. *OD* right eye, *OS* left eye

His corrected visual acuity was 6/7.4 right eye (OD; –5.25/–6.50×30) and 6/9 left eye (OS; –2.00/–4.00×95) with irregular astigmatism. There was evidence of periocular dermatitis with mild scaling and lichenification. Papillae were present on the upper tarsal conjunctivae, typical of allergic conjunctivitis. There were no other ocular signs of atopic disease. His intraocular pressure was 15 mmHg for both eyes (OU). Bilateral, asymmetrical inferior iridoschisis was present without corectopia or ectropion uveae (Fig. 1a, b). His anterior chamber was deep. Gonioscopy identified an open drainage angle allowing the ciliary body to be visualized through 360 degrees OU (Fig. 1c). Scheimpflug imaging measured an anterior chamber depth of 3.48 mm OD and 3.45 mm OS. Corneal topography documented bilateral keratoconus (Fig. 1d, e). A posterior segment examination revealed a healthy optic disc and macula.

There was no family history of keratoconus or iridoschisis.

## Discussion

There are only two previous cases reporting the rare combination of iridoschisis and keratoconus [4, 5]. The authors of these reports struggled to identify a unifying mechanism of keratoconus and iridoschisis, suggesting the unlikely hypotheses of a potential genetic association or common embryological derivation of involved tissues. Eye rubbing was not commented on specifically in such reports; it was not known to be strongly associated with keratoconus at the time. This report suggests, for the reasons outlined below, that eye rubbing may represent the common origin of coexistent keratoconus and iridoschisis.

Over the past 60 years since the term iridoschisis was first proposed by Loewenstein and Foster [6], numerous case reports and several case series have attempted to identify a common pathogenesis of iridoschisis from a variety of reported etiologies. Primary angle closure is strongly associated with iridoschisis [1, 7–9], and may be associated with glaucoma in approximately two-thirds of patients. One series identified complete or partial angle closure in 12 consecutive patients with iridoschisis leading to the conclusion that symptomatic or subclinical intraocular pressure spikes were contributory to its development [1]. The association of iridoschisis and plateau iris syndrome has recently been described in a young patient [10]. Gonioscopy and Scheimpflug imaging excluded primary angle closure in our patient. Trauma was considered a contributory factor in the development of iridoschisis by Loewenstein 1948 [11] and was supported by a subsequent report by Salmon [2]. In a similar fashion to primary angle closure, post-traumatic intraocular pressure peaks may result in shearing along dilator fibers with consequent splitting of the iris stroma [9, 11]. Subluxation of the crystalline lens resulting in adjacent sectoral angle closure and iridoschisis has been reported [12], with mechanical trauma from a mobile crystalline lens suggested to be contributing to its development. The possibility of intraocular pressure peaks in the setting of lens subluxation due to a secondary angle closure mechanism suggests similarities in pathogenesis in patients with primary angle closure. In summary, peaks in intraocular pressure in association with iris trauma are the most consistent themes in previous reports of iridoschisis. Chronic eye rubbing combines these insults to the iris: mechanical trauma and intraocular pressure spikes, which over years may conceivably have contributed to the development of iridoschisis in this case.

Ocular manifestations of atopic dermatitis include periocular dermatitis, atopic keratoconjunctivitis, atopic cataract, retinal detachment, and rarely, cicatricial lid disease (symblepharon, entropion, and so on).

Keratoconus is strongly associated with habitual eye rubbing. Multivariate analysis identified eye rubbing as the only statistically significant risk factor for keratoconus, after adjustment for atopy, among all commonly perceived risk factors [13]. Our patient was right hand dominant and the keratoconus was more pronounced in his right eye, compared to the left. A significant relationship between severe eye rubbing and keratoconus on the side of hand dominance has previously been demonstrated [14].

## Conclusions

In conclusion, this patient had keratoconus, iridoschisis, and severe allergic eye disease. This amalgamation of signs, particularly obvious in his right eye, might be explained on the basis of habitual eye rubbing. His behavior has been attenuated by treating the allergic eye disease.
